# Mentorship as a Mechanism for Equity, Retention & Scientific Innovation in Neuroscience-related Careers

**DOI:** 10.1093/oons/kvag005

**Published:** 2026-06-19

**Authors:** Bethlehem A Bekele, Zaki Alaoui, Ngum Agnes Mofor, Adejoke Elizabeth Memudu

**Affiliations:** Department of Cell Biology, Emory University, 615 Michael St, Atlanta, GA 30033, United States; Department of Neurobiology, Stanford University, 299 Campus Drive, Stanford, CA 94305, United States; Department of Neuroscience, Physiology and Pharmacology, University College London, Gower Street, London WC1E 6BT, United Kingdom; Department of Anatomy, Edo State University Iyamho, Along Km 007 Auchi Abuja Expressway Iyamho, PMB 04, 312102, Iyamho, Edo State, Nigeria

**Keywords:** equitable mentorship, Black neuroscientists, career development, retention in neuroscience, collective mentoring, structural equity

## Abstract

Despite rapid advances in neuroscience, Black scientists remain persistently underrepresented across training and career stages, reflecting structural inequities in mentorship, funding, and access to professional networks. These disparities limit not only individual career trajectories but also the formation of a strong scientific identity, thereby constraining the field’s capacity for innovation and global impact. In this perspective, we examine equitable mentorship as a critical tool for improving representation, retention, and scientific productivity in neuroscience. We define equitable mentorship as intentional, culturally responsive, and structurally supported guidance that extends beyond technical training to include career navigation, identity-affirming support, and access to networks. Drawing on evidence from the literature and personal experiences with mentorship models such as the Black in Neuro Mentorship Program, we highlight the value of collective, affinity-based, and cross-institutional approaches that distribute mentorship labor, foster belonging, and reduce the disproportionate burden placed on Black faculty and trainees. We argue that mentorship should be recognized as essential infrastructure for the neuroscience enterprise and call for institutional investment in structured, compensated mentorship programs to support Black neuroscientists and build a more equitable and innovative global workforce.

## Introduction

Neuroscience stands at a critical juncture, as one of the most rapidly evolving scientific disciplines. The field demands a diverse and innovative workforce capable of addressing complex questions about neural function, disease, and behavior. However, despite calls for systemic change, neuroscience continues to face persistent disparities in representation, retention, and opportunity. For example, Black trainees account for only 6% of neuroscience PhD students in the United States of America (USA), despite comprising 14.7% of the population. In the UK, although Black individuals comprise approximately 3% of the population, they represent only 1.2% of UK Research and Innovation (UKRI)–funded postgraduate researchers, revealing a significant underrepresentation at the most resourced levels of academic training. According to the British Neuroscience Association (BNA), this uneven representation trickles down through academic stages, with the percentage of early-career Black researchers reduced to 2% and effectively down to 0% for established researchers (Singleton et al. 2021, The Broken Pipeline Report – Leading Routes n.d., British Neuroscience Association 2022). Black scientists remain significantly underrepresented in faculty positions, with women, first-generation scholars, and researchers from low- and middle-income countries facing parallel burdens (National Academies of Sciences, Engineering, and Medicine; Health and Medicine Division; Board on Health Sciences Policy; Forum on Neuroscience and Nervous System Disorders 2021, Taffe and Gilpin 2021). These disparities reflect broader systemic barriers in research funding, in which Black scientists face significant disadvantages in grant success rates, even when controlling for factors such as publication record, institutional prestige, and prior funding history (Taffe and Gilpin 2021, Ginther et al. 2011). Addressing these disparities requires interventions at the structural level, with mentorship emerging as a key mechanism to improve both representation and retention.

The lack of diversity in neuroscience begins in the educational pipeline. Limited K-12 exposure to the field reduces later entry, particularly for historically marginalized students (Minen et al. 2023). In the U.S., Underrepresented Minority (URM) students earned 21% of bachelor’s degrees in 2012 but only 8.5% of STEM PhDs, with notably lower doctoral completion rates for Black students (Sowell et al. 2015). The UK shows similar patterns: only 4% of British Neuroscience Association (BNA) doctoral students and less than 1% of early career researchers identify as Black (British Neuroscience Association 2022). These statistics reflect persistent systemic barriers that create a ‘leaky pipeline’ and perpetuate underrepresentation at all career levels. Addressing these disparities requires systemic intervention, with equitable mentorship emerging as a critical mechanism for retention and advancement.

Equitable mentorship, defined as intentional and culturally responsive guidance that provides trainees with access to essential resources, networks, and sustained support, has emerged as a critical mechanism for addressing these disparities (Dualeh and Ibrahim 2024). The global need for such approaches is urgent. As the discipline encompasses vastly different methodological approaches and research questions, building a sustainable workforce requires not only training more scientists but ensuring that talent from all backgrounds can access and thrive in these evolving career pathways (Singleton et al. 2021).

A single graduate student or principal investigator cannot provide all the guidance a trainee needs to succeed in neuroscience. While a lab mentor may teach technical skills specific to their subdiscipline, trainees also need career mentorship to navigate funding mechanisms, build professional networks, and address challenges related to identity and belonging (Committee on Effective Mentoring in STEMM, Board on Higher Education and Workforce, Policy and Global Affairs 2019). Critically, psychological research demonstrates that seeing people who share one’s identity in academic and career settings leads to positive outcomes, including stronger academic identity formation and an enhanced sense of belonging (Zirkel 2002). Collective mentoring, affinity-based groups like Black In Neuro, and cross-institutional networks, such as NeuroMatch, uniquely provide these critical connections while also addressing the disproportionate burden that diversity, equity, and inclusion work places on underrepresented faculty and trainees (Murray et al. 2021).

**Figure 1:**
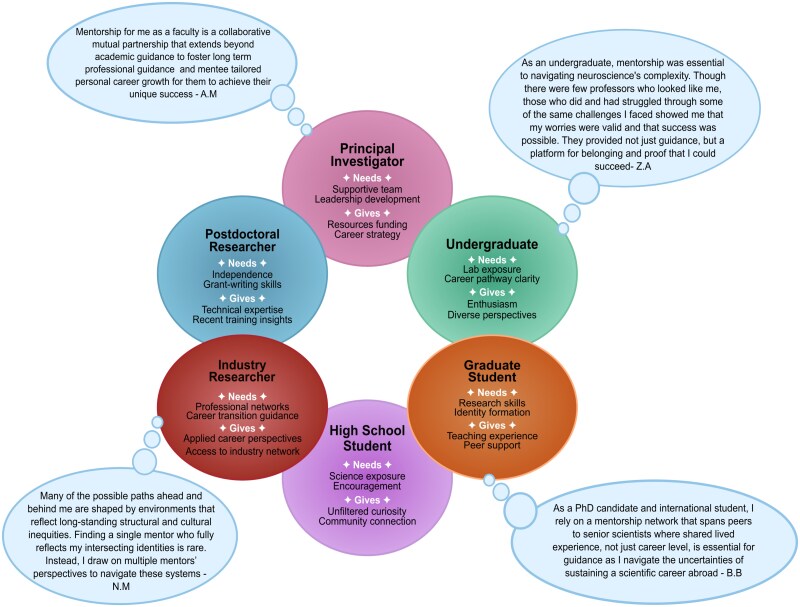
Mentorship as a distributed, reciprocal ecosystem across career stages. This schematic illustrates mentorship as a multi-directional and collaborative network spanning key stages of the scientific pipeline, including high school students, undergraduates, graduate students, postdoctoral researchers, industry scientists, and principal investigators. Each node highlights both the needs and contributions unique to that career stage, emphasizing mentorship as a reciprocal exchange rather than a unidirectional transfer of knowledge. Surrounding the central framework are quotes capturing mentorship experiences of the authors who are neuroscientists at different career stages.

This perspective examines mentorship as a critical tool for equity, retention, and innovation in neuroscience-related careers. We analyze existing barriers to equitable mentorship, explore how mentorship influences career trajectories and scientific productivity, and highlight successful models, including the Black in Neuro Mentorship Program. Guided by a conceptual framework in Fig. 1, which depicts mentorship as a distributed, reciprocal ecosystem across career stages, we illustrate how mentorship functions beyond traditional dyadic models to include peer, near-peer, and community-based support structures. By framing culturally inclusive mentorship as essential infrastructure for scientific progress, we call for institutional investment in structured, compensated mentorship programs to build a sustainable, equitable neuroscience workforce globally.

## Barriers and challenges in academia and research

Historical biases, discriminatory practices, and uneven access to resources have created systemic barriers that disproportionately affect women, racial/ethnic minorities, and those from economically disadvantaged backgrounds. Limited representation in leadership positions reinforces this; without same-identity role models or mentors, early-career researchers from these groups face higher rates of microaggressions and feelings of isolation. These challenges make it difficult to navigate hidden curricula (unspoken expectations and cultural codes) and informal networks that shape academic success (Garcini et al. 2022). A recent analysis of neuroscience faculty across 12 countries found that women are well represented among students and junior faculty but remain significantly underrepresented in senior faculty and department chair positions. This apparent ‘bottleneck’ effect highlights how, even when recruitment of diverse students improves, structural barriers, bias in advancement, and cumulative burdens perpetuate underrepresentation and inequity (Hamada et al. 2025).

Furthermore, findings from the Doctoral Initiative on Minority Attrition and Completion (Sowell et al. 2015) reveal that a majority of Black and Hispanic doctoral students feel integrated, supported, and treated equitably within their programs, with most disagreeing that they have experienced racism or differential treatment. However, beneath this generally positive climate, many still face significant structural and psychosocial pressures that threaten persistence. More than half of Black (58%) and three-quarters of Hispanic (75%) students reported losing interest in their field, and large proportions struggled with financial burden, family responsibilities, and mental or physical health concerns.

Findings from the British Neuroscience Association echo these disparities and ‘leaky pipeline’ for ethnic minority scientists in the UK. Inequities also manifest in academic recognition: a citation analysis of 33 934 neuroscience articles revealed that papers authored by researchers of color are under-cited relative to their presence in the field and has worsened in the last 25 years, particularly for women of color (British Neuroscience Association 2022). These implicit biases extend to funding and workload, where Black researchers receive fewer grants and as many minority academics shoulder disproportionate ‘diversity labor’ (British Neuroscience Association 2022, Hill-Jarrett et al. 2023). Such inequities can have direct effects on well-being, contributing to imposter syndrome, stereotype threat, and poorer mental health among underrepresented scholars.

Together, these findings suggest that while overt exclusion may be declining, the persistence of structural inequities, limited mentorship, and under-recognition of diverse contributions continues to erode the engagement, retention, and individual scientific identities in neuroscience. Such inequitable conditions in research create a self-reinforcing cycle: underrepresentation at advanced levels reduces visible pathways into leadership, leaving fewer mentors and advocates for URM trainees, and perpetuating the same barriers that hindered earlier generations.

## Mentorship initiatives to combat inequity

Developing and strengthening equitable mentorship is, therefore, a key strategy to counteract these inequities and improve retention and success of underrepresented groups in neuroscience. Equitable mentorship can act as a social mechanism to counterbalance the effects of isolation, bias, and exclusion by giving underrepresented early-career researchers access to networks, sponsorships, and culturally relevant guidance (Hill-Jarrett et al. 2023). Studies consistently demonstrate that structured and culturally responsive mentorship improves both objective outcomes (e.g. research productivity and completion rates) and psychosocial ones (e.g. belonging and scientific identity) (Bielczyk et al. 2019).

Unfortunately, early-career neuroscientists from marginalized groups seldom find mentors with shared complex and intersectional identities due to the scarcity of senior scientists from those backgrounds. This gap can perpetuate a power imbalance or a feeling of being misunderstood. Several initiatives exemplify how mentorship can directly promote equity in neuroscience (Hill-Jarrett et al. 2023). In an open letter to mentors of Black neuroscientists, researchers noted that strong mentoring ‘provides opportunities to publish, present, co-author grants, and develop networks.’ At the same time, negative mentorship ‘destroys confidence and fosters self-doubt,’ contributing to the leaky pipeline (Singleton et al. 2021). Similarly, the NIH-funded Diversifying the Community of Neuroscience (CNS) program, a longitudinal mentorship and training initiative aimed at increasing diversity in the neurosciences, demonstrated that sustained, cohort-based mentorship helps build a sense of community as participants reported greater confidence, retention, and persistence in neuroscience careers. Providing evidence that mentorship is most effective when designed as a tailored but evolving, developmental relationship rather than a one-off pairing (National Academies of Sciences, Engineering, and Medicine; Health and Medicine Division; Board on Health Sciences Policy; Forum on Neuroscience and Nervous System Disorders 2021).

Mentorship can serve as a powerful lever for advancing global equity in neuroscience, particularly in low-income countries where disparities in access to training, infrastructure and professional networks are a significant concern (National Academies of Sciences, Engineering, and Medicine; Health and Medicine Division; Board on Health Sciences Policy; Forum on Neuroscience and Nervous System Disorders 2021, Geminiani et al. 2024). In recent years, digital platforms have made it possible to mitigate structural barriers by making mentorship across countries possible. A compelling example is the International Online Mentoring Programme launched in 2017 by the Organization for Human Brain Mapping (OHBM), an organization dedicated to non-invasive neuroimaging research. By leveraging virtual communication to pair mentors and mentees across the world, the program expanded access to professional development and networking opportunities. Within its first four rounds, the program attracted over 1000 participants globally (Mentoring Programme n.d.). Notably, early-career principal investigators (PIs) also had the opportunity to be mentored by more established investigators and over 25% of participants served in dual mentor-mentee roles, reflecting strong cross-career reciprocity (Fig. 1). The program received positive feedback, with mentees reporting increased awareness of career pathways in neuroscience and an average satisfaction score of 4.06 out of 5. This outcome led to the OHBM Council’s decision to double the program’s mentoring budget and illustrates the need for continued institutional expansion of digital mentorship platforms (Bielczyk et al. 2019).

## Mentorship as a driver of scientific innovation in neuroscience

While neuroscience’s rapid advancement over the past several decades has been driven by its increasing interdisciplinarity, this alone does not automatically yield innovation. Instead, mentorship functions as a critical causal mechanism that enables scientific progress by helping trainees integrate disparate methodologies, languages, and conceptual frameworks. As modern neuroscience increasingly relies on large, complex datasets generated by high-resolution electrophysiology and imaging, mentors through programs such as NeuorMatch, play a pivotal role in guiding early-career researchers toward computational tools, machine learning approaches, and collaborative networks that would otherwise remain inaccessible. Even in institutions with formal cross-departmental training, mentorship bridges structural gaps by directing trainees to workshops, conferences, and interdisciplinary communities where novel ideas emerge. Large-scale efforts such as the Human Brain Project (HBP) demonstrate how mentorship can actively generate innovation. Through a deliberate ‘bottom-up’ model that empowered early-career researchers to organize conferences and shape collaborative projects, the HBP accelerated cross-disciplinary exchange and fostered new scientific directions (Geminiani et al. 2024). Similarly, in the USA where neuroscience PhD training has expanded more rapidly than in any other biomedical field, initiatives such as the NIH BRAIN Initiative underscore mentorship and cross-career collaboration not as ancillary supports but as essential drivers of discovery in an increasingly complex, data-driven ecosystem.

Academic institutions are among the most productive engines of scientific innovation worldwide, generating a substantial share of high-quality intellectual property (IP) that later serves as the basis for novel therapeutics, diagnostics, neurotechnology, and start-ups. The Association of University Technology Managers (AUTM), a membership-based organization for university technology transfer professionals, reported that over 7000 patents were granted to U.S. universities in both 2019 and 2020, highlighting a sustained academic patenting activity (Statistics (NCSES) NC for S and E 2024). These IPs give the innovators commercial rights and the ability to monetize their discovery. This growth is particularly evident in neuroscience, where UNESCO reported a 20-fold increase in patent filings between 2000 and 2021 (*Unveiling the Neurotechnology Landscape. Scientific Advancements Innovations and Major Trends*  2023). Key sectors in neuroscience, like neuromodulation, neuroimaging and AI-based behavioral tracking tools continue to reshape the intellectual property landscape (Sabnis et al. 2025). For example, the global neuromodulation devices market is valued at 9.54 billion US dollars and projected to grow to 25.43 billion US dollars by 2035 (Agrawal n.d.). This rapid commercial expansion reflects both the scientific momentum and the rising economic value of neuroscience-driven technologies.

However, meaningful participation in this ecosystem is not automatic; it requires the mentorship structures that give trainees the independence, expertise, and conceptual boldness needed to generate patentable innovation. Effective mentors help trainees identify novel questions, understand unmet clinical or technological needs, refine ideas into rigorous research plans, and navigate processes such as invention disclosure, patent filing, or translational collaboration with tech transfer offices. Without such mentor-mediated scaffolding, most potentially transformative ideas in neuroscience would never advance into high-quality IP or translational outcomes. In this way, mentorship serves not only as academic guidance but also as an accelerator, transforming scientific curiosity into tangible innovations with societal impact.

A key element of scientific innovation is not only making discoveries but also communicating them effectively so they can influence broader scientific discourse, attract collaborators, and translate into societal impact. Quality mentorship plays a pivotal role here, because strong scientific communication skills, including writing clear manuscripts, delivering compelling talks, and articulating the importance of findings are often learned through mentor modeling and explicit feedback. Programs such as the *Scientific Communication Advances Research Excellence* (SCOARE) initiative recognize this by training faculty mentors across multiple institutions to intentionally address scientific communication skill development as part of trainee development, with the ultimate goal of increasing research career persistence and success (Dahlstrom et al. 2022). This is particularly important in neuroscience, where communicating complex interdisciplinary findings clearly can accelerate uptake, collaboration, and impact. In this way, mentorship that emphasizes scientific communication, publication strategy, and translational thinking becomes a critical driver of innovation, enabling trainees to transform novel findings into high-impact papers, patents, and technologies that advance both the field and public understanding.

## Designing and evaluating effective mentorship programs in neuroscience

Despite increasing recognition of mentorship as a critical driver of equity, retention, and success in neuroscience, there remains limited empirical research on mentorship practices that effectively support diverse neuroscientists across training stages and career levels (Hill et al. 2022). Current mentorship structures in neuroscience primarily consist of traditional one-on-one models and team- or group-based approaches (Esplen et al. 2025), each offering distinct advantages and limitations that must be considered in program design. The traditional one-on-one mentorship model pairs an early-career trainee with an experienced researcher who provides scientific guidance, career advice, and professional networking opportunities (Fowler 2023). When mentor–mentee matching is based on shared research interests, values, and career goals, this model can support deep, personalized engagement and effective skill transfer (Chan et al. 2025). However, reliance on a single mentor may limit exposure to diverse perspectives and can exacerbate power imbalances, particularly for trainees from underrepresented backgrounds.

Team and group-based mentorship models address these limitations by distributing mentorship across multiple mentors and peers. These models include peer mentoring among trainees at similar career stages, senior mentor–cohort structures, and interdisciplinary research teams (Kim et al. 2025). Group-based mentoring promotes peer learning, collaboration, and social integration, fostering a sense of belonging that is particularly beneficial for neurodivergent trainees and those navigating identity-related challenges in neuroscience. The Summer of Translational Aging Research for Undergraduates (STAR U) program exemplifies this approach, combining structured mentorship with cohort-based learning to enhance diversity and research engagement in aging neuroscience (Esplen et al. 2025).

Effective mentorship programs require intentional design grounded in clear competency frameworks. A foundational element is strategic mentor–mentee matching aligned with research interests and career goals, alongside classification by career stage to ensure stage-appropriate support. Moreover, context-specific adaptations that align mentorship content with a country’s specific workforce needs are essential. For example, neurosurgery mentorship programs in several African countries deliberately prioritize neuroanatomical training and surgical skill development to address shortages in neurosurgery specialists (Nkenfou et al. 2025). In contrast, countries with expanding research infrastructure but limited funding mechanisms may prioritize mentorship in grant writing or international collaboration. Accordingly, mentorship programs should be designed with local infrastructure, cultural context, and regional workforce gaps in mind to ensure that global calls for inclusive mentorship translate into locally meaningful and actionable support. This can be achieved through careful assessment of regional needs prior to program design, partnerships with local institutions and professional societies, as well as the inclusion of local advisors who understand the specific barriers trainees face.

Rigorous evaluation of mentor competencies further strengthens program effectiveness. Mentors should demonstrate relevant research expertise, experience with appropriate animal or human models, and evidence of career progression, including grant-writing capacity (Kim et al. 2025, Bain et al. 2023, Nakanjako et al. 2011). Structured mentor training in inclusive communication, DEI principles, conflict resolution, emotional intelligence, research integrity, and goal setting improves mentor self-efficacy and mentee satisfaction (Esplen et al. 2025, Chan et al. 2025, Guy et al. 2025). Programs such as the Dana Foundation Career Network in Neuroscience & Society illustrate how mentor training can broaden trainees’ exposure to diverse neuroscience career pathways (Dana Foundation Career Network in Neuroscience and Society et al. 2025, Lambert 2025). Moreover, evaluation of mentorship programs should incorporate measurable short-, intermediate-, and long-term outcomes using both qualitative and quantitative methods, including surveys, interviews, focus groups, and progress tracking (Haberer et al. 2025, Hummel and Hersey 2023). Short-term outcomes include skill acquisition and mentee satisfaction; intermediate outcomes capture research productivity, conference participation, and fellowship attainment; and long-term outcomes assess career advancement, retention, leadership development, and sustained contributions to neuroscience (Hummel and Hersey 2023). Collectively, evidence demonstrates that structured, competency-driven mentorship programs enhance equity, retention, publication success, grant acquisition, and scientific innovation within neuroscience (Azevedo et al. 2023, Crites et al. 2023).

## Mentorship in action: case studies supporting Black neuroscientists

Addressing disparities in neuroscience careers requires intentionally designed mentorship programs that support Black trainees globally and operate beyond traditional, institution-bound models. Several initiatives, including Black In Neuro, International Brain Research Organization (IBRO), and TReND in Africa, and targeted mentorship efforts for women through World Women in Neuroscience, offer complementary approaches to addressing these systemic gaps (Bain et al. 2023, Haberer et al. 2025).

Black In Neuro (BIN) represents a leading model for community-centered mentorship tailored to Black neuroscientists. Founded to address persistent inequities in representation, funding access, and professional visibility, Black In Neuro is a global network dedicated to empowering Black scholars and professionals across neuroscience-related fields (Murray et al. 2021, Smith et al. 2022). Its mission to ‘diversify the neurosciences by building a community that celebrates and supports Black scholars worldwide’ explicitly positions mentorship as infrastructure for equity and scientific innovation. BIN’s mentorship program spans undergraduate, graduate, postdoctoral, early-career, and senior scientists and engages non-Black allies committed to advancing equity in neuroscience. Through structured and individualized mentor–mentee matching, career development programming, and affinity-based community building, Black In Neuro addresses well-documented barriers faced by racially minoritized scientists, including inequities in funding priority, collaborative opportunities, and professional networks (Singleton et al. 2021, Davis et al. 2022). Importantly, BIN’s model distributes mentorship responsibilities across a collective network (Fig. 1), reducing the disproportionate mentorship burden often placed on a small number of Black faculty while fostering peer support, visibility, and long-term retention within the field (Murray et al. 2021). By centering identity-affirming mentorship and global accessibility, Black In Neuro demonstrates how structured, community-driven mentorship can counter systemic exclusion while strengthening the neuroscience workforce.

Beyond BIN, international initiatives such as IBRO and TReND in Africa play a critical role in expanding mentorship and training opportunities for underrepresented neuroscientists, particularly across low- and middle-income countries. IBRO has established regional education and training programs, including Africa-focused schools and mentorship grants, aimed at building capacity, fostering collaboration, and advancing neuroscience education worldwide (Bain et al. 2023). These initiatives are especially relevant given Africa’s disproportionate burden of neurological disease alongside shortages of skilled researchers, limited funding, and restricted access to mentorship (Ng’oda et al. 2024). IBRO-supported programs provide advanced coursework, hands-on laboratory training, and sustained mentorship in areas such as neurodegeneration and translational neuroscience, contributing to improved research output, career satisfaction, and faculty retention (Ashley et al. 2023, Mohamed et al. 2025, Van Dongen et al. 2024).

Similarly, TReND in Africa (Teaching and Research in Natural Sciences for Development in Africa) addresses critical gaps in neuroscience capacity by offering training and mentorship in neurogenetics, molecular biology, computational neuroscience, bioinformatics, genome editing, and open hardware (Baden et al. 2020). Through initiatives such as TReND-CaMINA, which focuses on computational neuroscience and machine learning, TReND mitigates systemic barriers to skill acquisition and research independence among early-career African neuroscientists (Nkenfou et al. 2025, Meier 2023). Together, IBRO and TReND exemplify international mentorship models that enhance global equity, research productivity, and long-term career sustainability for Black neuroscientists (Dualeh and Ibrahim 2024, Waljee et al. 2020).

Complementing these efforts, World Women in Neuroscience (WWN) highlights the importance of targeted mentorship for other underrepresented groups in STEM, particularly women and Black women in neuroscience. Despite growing attention to gender equity, representation of Black women declines sharply with increasing academic rank (Menifield et al. 2024, Morgan et al. 2021). WWN addresses this gap through structured, year-long mentorship programs that pair mentees with trained senior mentors based on career stage and research interests, providing both professional guidance and psychosocial support during critical career transitions (Alshayhan et al. 2023, Farkas et al. 2019). WWN’s partnership with IBRO through the Global Connectome Mentoring and Networking Program further illustrates how cross-organizational collaboration can amplify mentorship impact at a global scale (Guy et al. 2025).

Collectively, these initiatives demonstrate that effective mentorship for Black neuroscientists and other underrepresented groups must be structured, identity-aware, globally accessible, and evaluated for long-term impact. Black In Neuro stands as a powerful example of how community-led mentorship can address inequities in representation, retention, and opportunity, reframing mentorship not as an informal practice but as essential infrastructure for an equitable and innovative neuroscience workforce.

## Conclusion and call to action

Traditional one-on-one mentorship models remain foundational in academic neuroscience, offering trainees access to scientific expertise, career guidance, and professional networks. However, when relied upon in isolation, these models are insufficient to meet the diverse and evolving needs of today’s neuroscience workforce. Power imbalances, cultural and generational differences, and the limited capacity of individual senior mentors can constrain open dialogue, reduce consistency of support, and unintentionally perpetuate inequities in training and career advancement, particularly for Black trainees and others from historically marginalized backgrounds. Together, these structural constraints can reduce transparency, fragment mentorship experiences, and unintentionally perpetuate inequities in neuroscience training and career advancement.

As emphasized throughout this perspective, equitable mentorship must extend beyond dyadic relationships to include collective, identity-affirming, and cross-institutional models that provide sustained access to resources, networks, and belonging. Programs such as affinity-based mentorship networks like the Black in Neuro mentorship program demonstrate how distributed mentorship can reduce the disproportionate mentorship burden placed on underrepresented faculty while improving retention, professional development, and scientific engagement across career stages.

We therefore call on institutions, funding agencies, professional societies, and journals to recognize mentorship as essential infrastructure for the neuroscience enterprise. This requires intentional investment in structured and compensated mentorship programs; formal recognition of mentorship labor in hiring, promotion, and funding decisions; and support for community-based and global mentoring networks that expand access beyond individual laboratories. By reimagining mentorship as a shared, resourced, and equity-centered responsibility, the neuroscience community can better support Black neuroscientists while creating environments that foster innovation, collaboration, and sustained excellence for all.

## Data Availability

No datasets were generated or analyzed during the current study. Data sharing is not applicable to this article.
